# Superior Comprehensive Mechanical Properties of a Low-Carbon Medium Manganese Steel for Replacing AISI 4330 Steel in the Oil and Gas Industry

**DOI:** 10.3390/ma16020490

**Published:** 2023-01-04

**Authors:** Xinjun Sun, Gang Liu, Xiaokai Liang, Shuai Tong

**Affiliations:** 1 Central Iron and Steel Research Institute Company Limited, Beijing 100081, China; 2 Innovation Research Institute for Carbon Neutrality, University of Science and Technology Beijing, Beijing 100083, China; 3 Beijing Advanced Innovation Center for Materials Genome Engineering, University of Science and Technology Beijing, Beijing 100083, China

**Keywords:** low-carbon medium manganese steel, AISI 4330 steel, reversed austenite, mechanical properties, fatigue crack growth rate

## Abstract

A low-carbon medium manganese steel (0.12C-3.13Mn) containing Cr, Ni, Mo, V, and Cu elements was designed to replace the AISI 4330 steel applied in the oil and gas industry. The mechanical properties, microstructures, and fatigue crack growth rate were comparatively analyzed using uniaxial tension tests, microstructure characterization, and compact tension with fatigue crack growth characterization. The results showed that the ductility and −40 °C impact energy of 0.12C-3.13Mn steel were better than AISI 4330 steel (from 115 J to 179 J), while the yield strength of 957 MPa of the former was lower than the latter of 1060 MPa after being subjected to the same tempering process. The microstructure of 0.12C-3.13Mn steel was composed of a mixture of tempered martensite, reversed austenite, and nanosized precipitation particles, while the microstructure of S4330 steel contained ferrite and large-size Fe_3_C with lath and near-spherical morphologies. Compared to Cr-rich Fe_3_C, (V, Mo)C and Cu-rich particles have smaller sizes and, thus, provide more strengthening increment, leading to a higher yield ratio. The impressive fatigue-resistance property was obtained in 0.12C-3.13Mn steel because the threshold value was 5.23 MPa*m^1/2^ compared to the value of 4.88 MPa*m^1/2^ for S4330 steel. Even if the fatigue crack grew, the stress intensity factor range of 0.12C-3.13Mn steel was obviously wider than that of AISI 4330 steel due to the presence of reversed austenite and secondary cracks. Overall, the AISI 4330 steel could be replaced with the designed 0.12C-3.13Mn steel due to the similar strength and better ductility, low-temperature toughness, and fatigue-resistance property.

## 1. Introduction

AISI 4330 steel (namely S4330 steel), which is a CrNiMoV low-alloy structural steel, has a wide application history in the aerospace industry, especially in rotor engine components [1]. In the past decades, S4330 steel has also shown potential for application in the oil and gas industry due to its good match of strength and toughness [2,3]. However, as the exploitation depth of oil and gas wells increased, superior low-temperature toughness and fatigue resistance properties were needed [4]. Ni was introduced into S4330 steel to increase the low-temperature toughness because it can promote cross slip of dislocations so that the deformation would continue [5]. The isothermal temperature of 500 °C was also used to induce the upper bainite transformation to improve the strength and toughness match and inhibit the fatigue crack growth [6,7]. The studies of “partially substituting vanadium for molybdenum” in S4330 low-alloy steel also showed that Mo-V steel has better hardenability than Mo steel [8].

However, nowadays, the traditional medium carbon S4330 steel can hardly meet the performance requirement for the more severe service environment during oil and gas exploitation due to the presence of large-size carbides. Therefore, it is necessary to optimize the alloying chemistry and develop a new-type low-alloy constructional steel. Decreasing the C content may be an effective way to improve the comprehensive properties, such as corrosion resistance, toughness, ductility, and so on [9,10,11]. Unfortunately, the strength would decrease as the carbon content decreases. It is noted that low-carbon medium manganese steel displays superior mechanical properties, potentially replacing S4330 steel [12]. Similarly, the yield strength of traditional medium manganese steel is usually low due to the low C content and the existence of reversed austenite (RA) [13]. The poor match of yield strength, low-temperature toughness, and fatigue-resistance property urgently need to be improved in traditional low-carbon medium manganese steel. There are several ways to reach this goal: the addition of microalloying elements to increase yield strength, the addition of alloying Mn to optimize RA transformation, and a proper heat treatment schedule to tailor the RA content.

Microalloying is an effective way to strengthen the matrix with little ductility and toughness loss [14]. Microalloying elements are also an effective way to refine the microstructure. V tends to precipitate at low temperatures due to the high solubility product with C compared with Nb and Ti, so the carbide has nanoscale size [15]. Although the V carbides are easiest to be coarsened, the addition of Mo shows the obvious effect of inhibiting the coarsening and increasing the hardenability [16]. Furthermore, it has been well documented that increasing Mn content was beneficial to introduce RA [17], and a small amount of RA can significantly increase the low-temperature toughness and fatigue-resistance property [18]. There are two ways to obtain a small amount of RA: long-time annealing at a relatively low temperature and short-time annealing at a relatively high temperature within the intercritical region (α + γ two-phase region). On the one hand, the C and Mn contents in austenite are low during high-temperature tempering causing RA to have low stability and leading to the negative transformation-induced plasticity (TRIP) effect [19]. On the other hand, dislocation recovery and recrystallization may occur during high-temperature tempering, causing low strength [20]. Thus, a long-time heat treatment at a relatively low temperature was selected. In this paper, a new low-carbon medium manganese steel with additions of V, Mo, Ni, and Cu was developed to replace traditional S4330 steel, and its microstructures and mechanical properties were investigated.

## 2. Materials and Methods

**Figure 1:**
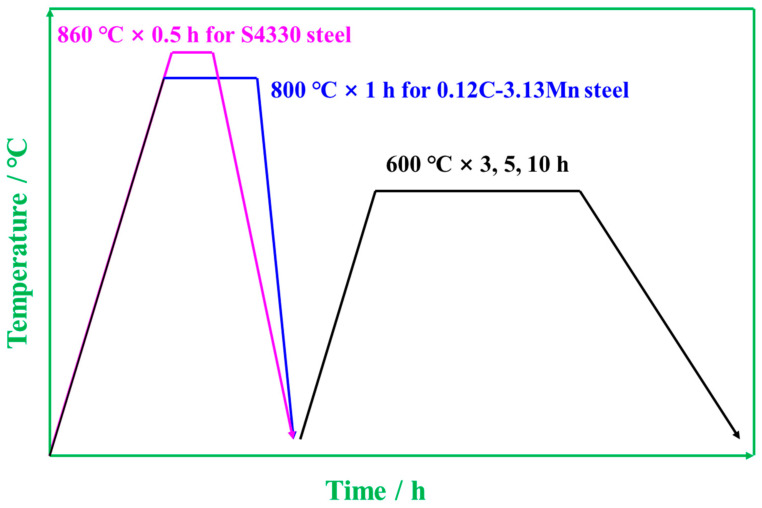
Schematic diagram for 0.12C-3.13Mn steel and S4330 steel.

Material Preparations: The nominal composition of the designed 0.12C-3.13Mn steel and S4330 steel are shown in Table 1. The 0.12C-3.13Mn steel was melted in a 100 kg high-frequency vacuum induction furnace and cast into a round bar billet with a diameter of 120 mm. The 120 mm round bar billet was further forged into a 25 mm round bar using several passes after being reheated at 1000 °C. Subsequently, the quenching and tempering (Q-T) treatments were carried out at temperatures of 800 °C and 600 °C, respectively. The A_c1_ and A_c3_ temperatures of 0.12C-3.13Mn steel were measured at about 592 and 791 °C using the thermal expansion method, so the microstructure of tempered martensite and RA could be obtained using the Q-T treatment. The S4330 steel samples were cut using the relative equipment in service. For S4330 steel, a quenching temperature of 860 °C and a tempering temperature of 600 °C were chosen, followed by oil cooling. The heat treatment process is shown in Figure 1.

Mechanical Property Tests: The round tensile samples, with a gauge length of 50 mm according to the standard of GB/T228.1-2010, were performed at a crosshead speed of 1 mm/min using a WE-300 hydraulic tensile testing machine (Jinan Chenxin testing machine Manufacturing Co., LTD., Jinan, China). The Charpy V-notch samples were machined according to the GB/T229-2007, which was performed using a JBN-300B impact machine at ±40 °C. The standard FCGR samples were machined following the GB/T 6398-2017. The sample thickness (B) was 12.5 mm, and the length and width were 62 mm.

Microstructure Characterization: After mechanical grinding and polishing, the samples were etched in a mixed solution of 4% nitric acid and 96% absolute ethyl alcohol. The prepared samples were observed using FEI (Hillsboro, OR, USA) Quanta 650 field emission scanning electron microscopy (SEM). After electrolytic polishing in a solution of 10% perchloric acid and 90% absolute ethyl alcohol, the samples were placed in an SEM equipped with the OXFORD NordlysNano detector (Oxford Instruments, Abingdon, England) for the electron backscattering diffraction (EBSD) test. The scanning step size was 0.15 μm, and the detecting area was about 100 μm × 100 μm. HKL-Channel 5 (Oxford Instruments, Abingdon, England) was used for postprocessing. Samples for transmission electron microscopy (TEM) observations were cut into slices of about 300 μm thickness using an electron discharge machine. The mechanically thinned slices were punched into several round disks with 3 mm diameter and then further thinned using a twin-jet electrolytic polishing machine under a constant current of 30 mA and a temperature of ~−20 °C. FEI (Hillsboro, OR, USA) TECNAI G^2^ 20 was used for the microstructure observation with an accelerating voltage of 200 kV. The X-ray diffraction (XRD) Bruker (Karlsruhe, Germany) D8 Advance instrument was used for the phase identification and the Co-Kα target was used to scan the diffraction peaks of phases. The content of RA strongly depends on the RA diffraction peaks of (200), (220), and (311) and the ferrite diffraction peaks of (200) and (211) [21]:

(1) $$V_{\gamma }=\frac{1.4I_{\gamma }}{I_{\alpha }+1.4I_{\gamma }}$$

where *I_α_* and *I_γ_* are the diffraction peaks of RA and ferrite, respectively. The dislocation density was further estimated using the modified Williamson–Hall method [22,23]:

(2) $$\Delta K≅\frac{0.9}{D}+bM\sqrt{\frac{\pi }{2}\rho }\left(K{\bar{C}}^{1/2}\right)$$

where *D* is the crystallite size, *K* is the magnitude of the diffraction vector, *b* is the magnitude of the Burgers vector, *ρ* is the dislocation density, $\bar{C}$ is the dislocation contrast factor, and *M* is a dimensionless constant.

## 3. Results

### 3.1. Mechanical Properties

**Figure 2:**
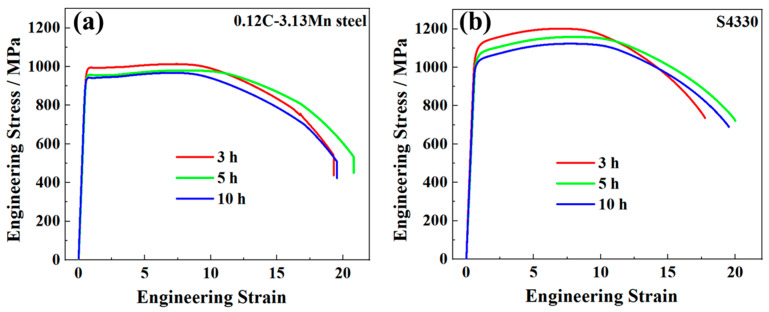
Engineering strain and stress curves of (**a**) 0.12C-3.13Mn steel and (**b**) S4330 steel.

The engineering strain and stress curves of 0.12C-3.13Mn steel and S4330 steel are displayed in Figure 2. It was found that 0.12C-3.13Mn steel had better total elongation (TE); however, the strength was relatively low. There were yield platforms in 0.12C-3.13Mn steel from 3 h to 5 h tempering at 600 °C (Figure 2a), and the low difference between yield strength and tensile strength potentially indicated that the yield strength was significantly increased by the introduced strengthening methods. No yield platforms were found in S4330 steel (Figure 2b).

**Figure 3:**
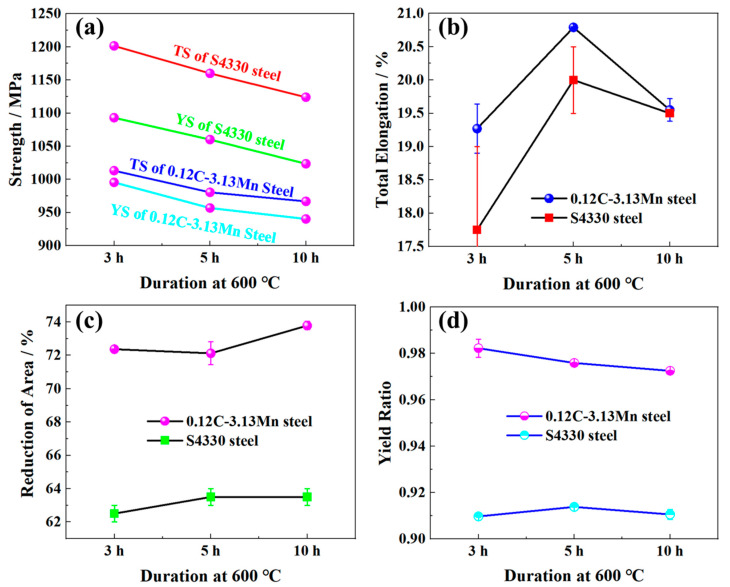
Mechanical properties of 0.12C-3.13Mn steel and S4330 steel: (**a**) yield strength and tensile strength; (**b**) total elongation; (**c**) reduction of area; (**d**) yield ratio.

Both the tensile strength (TS) and yield strength (YS) decreased as the tempering time increased. From Figure 3a, the TS and YS of S4330 steel are about 150 MPa and 100 MPa higher than those of 0.12C-3.13Mn steel subjected to the same tempering time, respectively. The TE of 0.12C-3.13Mn steel was slightly superior to that of S4330 steel, and both types of steel had the best total elongation (TE) after being tempered at 600 °C for 5 h (Figure 3b). Compared to TE, the reduction of area of 0.12C-3.13Mn steel (about 72%) was superior to that of S4330 steel (about 63%) (Figure 3c). The yield ratio was more than 0.97 for the 0.12C-3.13Mn steel, while it was lower than 0.92 for the S4330 steel (Figure 3d).

**Figure 4:**
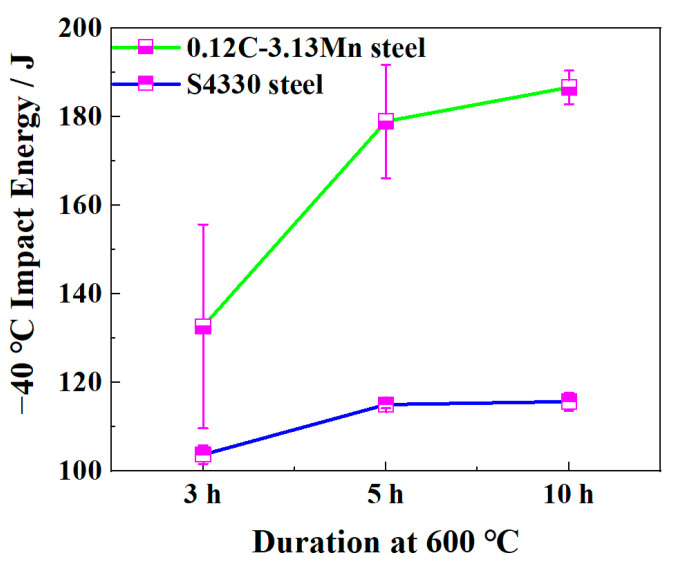
−40 °C impact energies of the 0.12C-3.13Mn steel and S4330 steel.

The 0.12C-3.13Mn steel had higher −40 °C impact energies than S4430 steel, showing better low-temperature toughness (Figure 4). The −40 °C impact energy increased significantly from 133 J after 3 h tempering to 179 J after 5 h tempering, then it increased slightly to 187 J after 10 h tempering. For S4330 steel, there was little difference in impact energy from 3 h to 10 h tempering.

### 3.2. Microstructure Characterization

**Figure 5:**
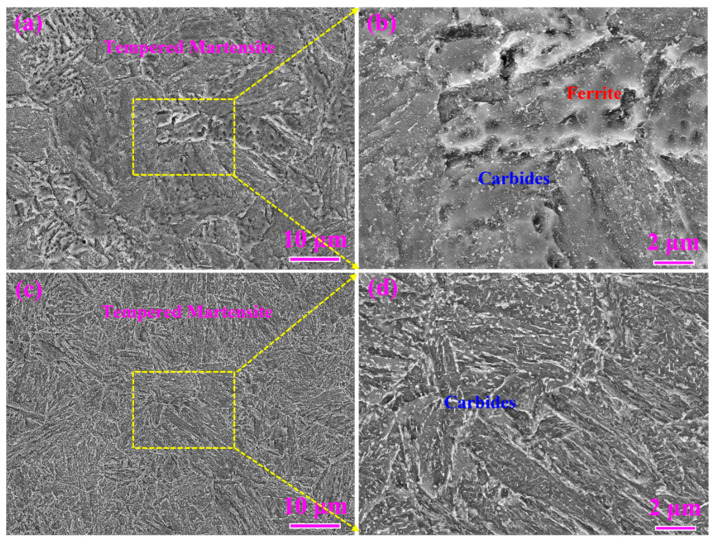
SEM images: (**a**,**b**) 0.12C-3.13Mn steel tempered at 600 °C for 5 h; (**c**,**d**) S4330 steel tempered at 600 °C for 5 h. ((**b**,**d**) are the magnified zones of (**a**,**b**), respectively).

The microstructures of both types of steel were tempered martensite based on the observation of SEM (Figure 5). The microstructure of S4330 steel was slightly finer than that of 0.12C-3.13Mn steel as seen in Figure 5a,c. There were numerous carbide particles distributed within the matrix. Therein, the size and volume fraction of carbides in 0.12C-3.13Mn steel were much lower than those in S4330 steel due to the higher carbon content in S4330 steel. The morphologies of carbides were also different in 0.12C-3.13Mn steel and S4330 steel: the former was spherical and the latter was rod-like and near-spherical (Figure 5b,d).

**Figure 6:**
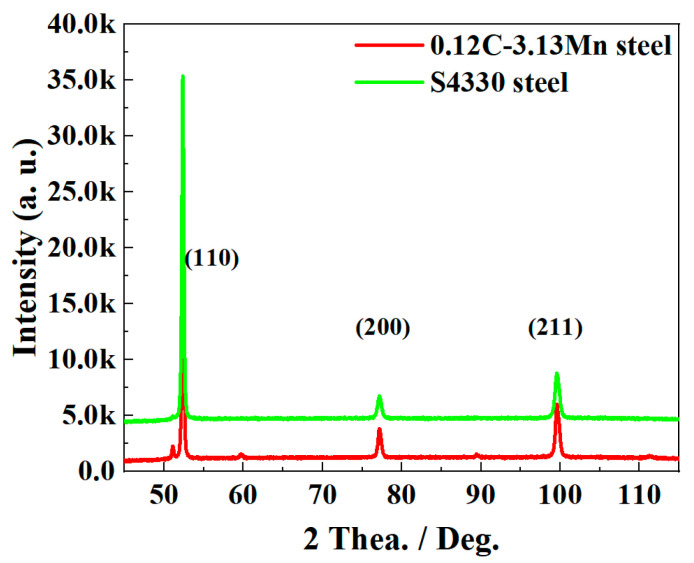
XRD profiles of 0.12C-3.13Mn steel and S4330 steel tempered at 600 °C for 5 h.

The RA diffraction peaks were detected in 0.12C-3.13Mn steel, while no RA diffraction peaks were detected in S4330 steel, as shown in Figure 6. The RA content in 0.12C-3.13Mn steel was calculated as 5.3%. In addition, the calculated dislocation densities were 7.168 × 10^14^ and 1.434 × 10^15^/m^2^, respectively.

**Figure 7:**
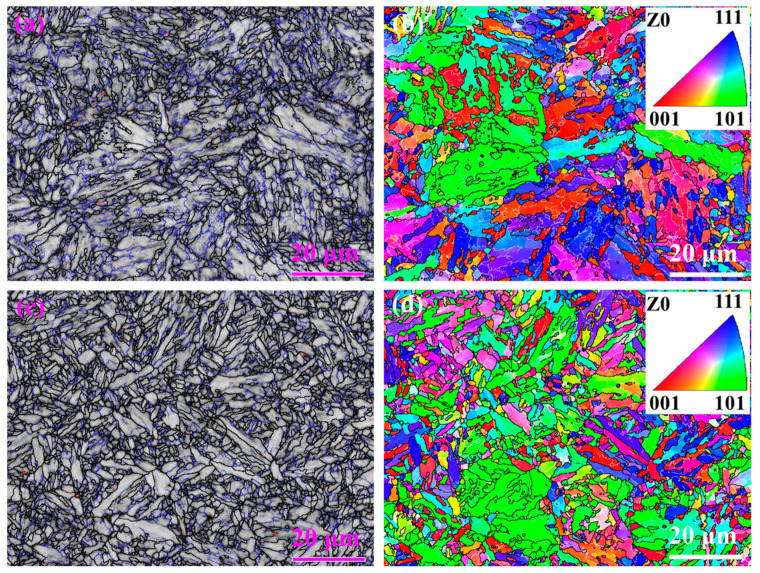
EBSD images: (**a**) Band Contract with RA marked and (**b**) Inverse pole figure (IPF) map of 0.12C-3.13Mn steel tempered at 600 °C for 5 h; (**c**) Band Contract with RA marked and (**d**) IPF map of S4430 steel tempered at 600 °C for 5 h. (The black line indicates high angle grain boundaries, while the fine blue line indicates low angle grain boundaries in (**a**,**c**). The black line indicates high angle grain boundaries, while the fine white line indicates low angle grain boundaries in (**b**,**d**)).

There was no obvious RA phase detected using EBSD, although, both bcc (α-Fe) and (γ-Fe) were selected during detection. There are two main reasons: on the one hand, the content of RA was only about 5.3%, so the RA size was small; on the other hand, the EBSD scanning step size was about 150 nm, which may be over the average RA size (Figure 7a). Figure 7a,b also shows that the density of blue and white lines was higher in 0.12C-3.13Mn steel than in that of S4330 steel, indicating that the level of recovery was higher in S4330 steel. In addition, there was no obvious texture in the two types of steel, as shown in Figure 7b,d.

**Figure 8:**
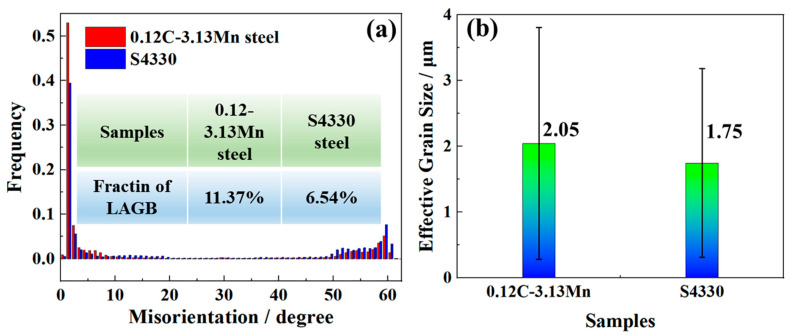
(**a**) Misorientation of 0.12C-3.13Mn steel and S4330 steel; (**b**) effective grain size of 0.12C-3.13Mn steel and S4330 steel.

The statistical information identified that the fraction of low-angle grain boundaries reached 11.37% in 0.12C-3.13Mn steel, while it was only 6.54% in S4330 steel (Figure 8a). In addition, the mean effective grain size was 2.05 and 1.75 μm for 0.12C-3.13Mn steel and S4330 steel, respectively (Figure 8b).

**Figure 9:**
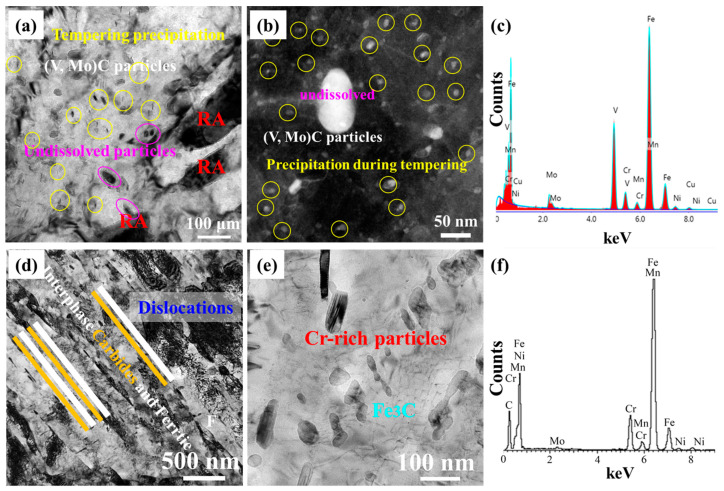
TEM images: (**a**) Bright filed image showing the precipitation particles and RA of 0.12C-3.13Mn steel; (**b**) STEM result showing precipitation particles and (**c**) TEM-EDS of particles; (**d**) Bright filed image showing the structure of martensitic lath and carbides; (**e**) Fe_3_C particles with different morphologies; (**f**) TEM-EDS of particles.

Figure 9 displays the TEM images of 0.12C-3.13Mn steel and S4330 steel. Two different sizes of microalloying precipitates, which was identified as (V, Mo)C using TEM-EDS in Figure 9c, were found (Figure 9a,b). The larger particles measuring tens of nanometers in size could be undissolved particles due to a relatively low reheating temperature of 800 °C, and the smaller particles measuring several nanometers in size could be particles precipitated during tempering at 600 °C. In addition, the RA was measured at about 130 nm in size. In S4330 steel, the tempering martensite, containing lath with high-density dislocations and carbides, was also observed using TEM (Figure 9d). The carbides were mainly Fe_3_C, where Cr and Mo accounted for a large proportion (Figure 9f). Therefore, the particles were the alloyed Fe_3_C, and the alloyed Fe_3_C particles had two morphologies: lath-like and near-spherical (Figure 9e).

### 3.3. Fatigue Crack Growth Rate

**Figure 10:**
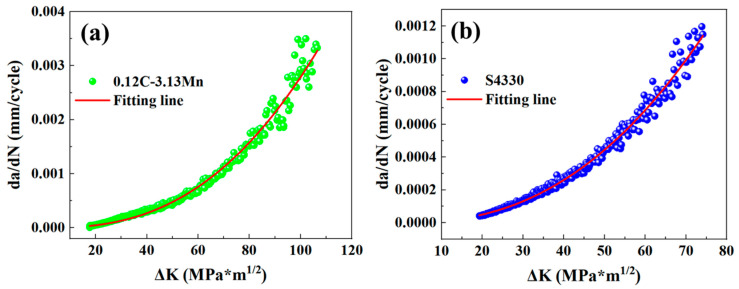
Fatigue crack growth rate curves for (**a**) 0.12C-3.13Mn steel and (**b**) S4330 steel.

The typical FCG rate (FCGR) curve contains three stages [24]. In the first stage, the fatigue crack does not grow until the range of the stress intensity factor range (Δ*K*) reaches a critical value, which is the threshold value (Δ*K_th_*), so this stage is also called the low-FCGR zone. The Δ*K_th_* is usually defined as the Δ*K* when the FCGR is 10^−7^ mm/cycle. In the second stage, the fatigue crack grows as the Δ*K* > Δ*K_th_*, and this is the stable FCG, where the FCGR and Δ*K* obey the Paris equation [25]:

(3) $$\frac{da}{dN}=C{\left(\Delta K\right)}^{m}$$

where, *a* is the crack size, *N* is the cycle, *C* and *m* are constants affected by temperature, humidity, medium, loading frequency, and so on. In the third stage, the FCGR will increase rapidly, and the FCG will enter the unstable growth stage until the final fracture. The second stage of the FCGR curve for the two types of steel is shown in Figure 10. The Δ*K* ranged from 17 to 107 MPa*m^1/2^ in 0.12C-3.13Mn steel (Figure 10a), while the Δ*K* ranged from 19 to 74 MPa*m^1/2^ in S4330 steel (Figure 10b). The FCGR ranged from 3.18 × 10^−5^ to 0.00330 mm/cycle in 0.12C-3.13Mn steel, while the FCGR ranged from 4.58 × 10^−5^ to 0.00114 mm/cycle in S4330 steel.

## 4. Discussions

### 4.1. “Medium Manganese Design” for Optimizing the RA Transformation

The RA transformation was significantly affected by the heat treatment schedule, initial microstructure, and forming process. However, these factors were limited for the steel applied in the oil and gas industry due to their enormous volume. The addition of C and Mn was an effective way to induce the RA transformation, which has been approved by the design strategy of medium manganese steel. It was noted that the yield strength of medium manganese steel would decrease as the RA content increased, although, there was an excellent production of tensile strength and total elongation [26,27]. Moreover, our previous work suggested that the low-temperature toughness first increased to maximum at low RA content and then decreased as the RA content further increased [14,18]. These results showed that RA should be tailored at low content to ensure a good match of yield strength and toughness. According to the phase transformation characteristics (A_c3_ temperature of 592 °C), the tempering temperature of 600 °C was selected to induce RA transformation.

**Figure 11:**
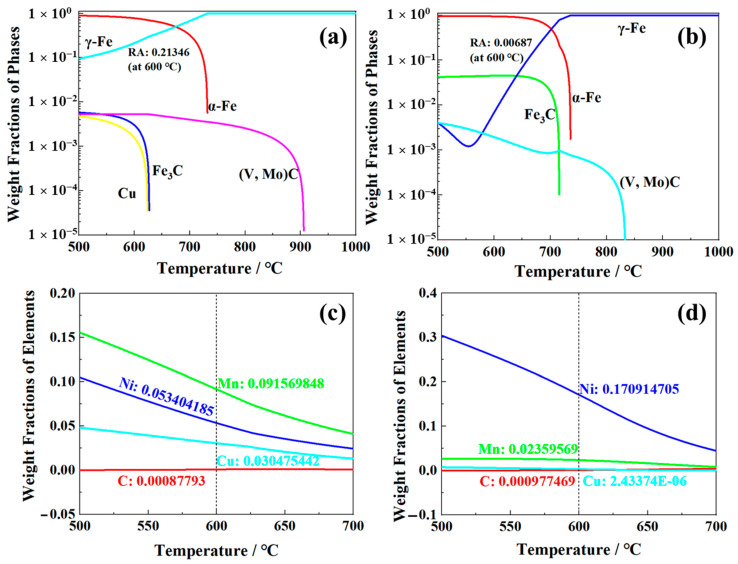
Thermo-Calc calculations: phase weight fractions of (**a**) 0.12C-3.13Mn steel and (**b**) S4330 steel; element weight fractions in RA of (**c**) 0.12C-3.13Mn steel and (**d**) S4330 steel, respectively.

The phase fractions varying with temperature are calculated using Thermo-Calc with database TCFE10.0, as shown in Figure 11. The RA content was about 0.7% in S4330 steel (Figure 11b), which was consistent with the detected result using XRD (Figure 6). For 0.12C-3.13Mn steel, the content of RA was about 21.3% (Figure 11a), which was higher than the result tested using XRD (Figure 6). The RA content lower than the equilibrium value calculated was mainly because the tempering time was much shorter than the time needed to reach equilibrium. Guo, et al. and Varanasi, et al. believed that the precipitates and microstructural defects were the main reason for restraining the RA transformation [28,29]. Guo, et al confirmed that the interface mobility changed when the moving phase boundary encounters some precipitates or microstructural defects. Varanasi, et al verified that the high solution drag pressure and Zenner pining pressure inhibited the RA transformation. Therefore, the (V, Mo)C particles were the main reason for the nonequilibrium RA transformation in 0.12C-3.13Mn steel.

C, Mn, Ni, and Cu are all the elements used to enlarge the austenite single-phase region and stabilize austenite. The calculated results showed that 0.12C-3.13Mn steel had higher RA forming ability than S4330 steel, attributed to the “substituting Mn for Ni” strategy. Excellent mechanical properties were obtained by Jiang, et al. using “substituting Mn for Ni” in alloy design in combination with the utilization of QLT heat treatment compared to traditional 5Ni steel [30]. Certainly, the role of Cu addition on RA formation is nonnegligible [31]. Furthermore, the chemical composition in the FCC phase (RA) of 0.12C-3.13Mn steel and S4330 steel was displayed in Figure 11c,d. There was similar C content in these two steels, but the contents of Mn and Cu in 0.12C-3.13Mn steel were higher than that in S4330 steel. It was noted that total contents of C, Mn, Ni, and Cu in the former steel were lower than that in the latter steel; however, the RA content was higher, potentially showing that Mn and Cu addition could replace Ni alloying to significantly improve the RA transformation.

The Ms temperature was the basic empirical equation, proposed by Capdevila, et al from a neural network analysis, used to evaluate the RA stability [32]:

(4) Ms(°C)=491.2−308C−30.6Mn−8.9Cr−16.6Ni+2.4Mo−11.3Cu−14.5Si

where C, Mn, Cr, Ni, Mo, Cu, and Si are the concentrations in RA (wt.%). The equation showed that the coefficient of Mn is highest among Cr, Ni, Cu, and Si, implying that Mn has the best ability to stabilize RA. Thus, the “substituting Mn for Ni” strategy is the effective and more cost-effective way to induce RA transformation and stabilize RA.

### 4.2. Precipitation Strengthening and Toughening Mechanism Analysis

It was the (V, Mo)C and Cu-rich particles that mainly provided the strengthening increment in 0.12C-3.13Mn steel. However, (V, Mo)C and Cu-rich particles usually induced different strengthening mechanisms according to the interaction between particles and dislocations. Generally, the dislocation line encountered (V, Mo)C particles by forming Orowan loops, while the dislocation line cut through Cu-rich particles [33]. In fact, the transition of strengthening mechanisms strongly depended on the critical particle size: the Orowan strengthening mechanism occurred when the precipitation size was over the critical particle size, or the cutting though strengthening appeared [34]. The critical size of (V, Mo)C precipitation was no more than 5 nm, while the critical size of Cu-rich particles reached tens of nanometers [35,36]. The synergistic strengthening effect of (V, Mo)C and Cu-rich particles obeyed the following equation:

(5) $$\sigma_{P}=\sqrt{\sigma_{\left(V,Mo\right)C}^{2}+\sigma_{Cu}^{2}}$$

where $\sigma_{\left(V,Mo\right)C}$ and $\sigma_{Cu}$ are the precipitation strengthening of (V, Mo)C and Cu-rich phase, respectively, which follow the equations [37,38]:

(6) $$\sigma_{\left(V,Mo\right)C}=\frac{0.8MGb}{2\pi \sqrt{1-\nu }l}\ln\left(\frac{d}{2.45b}\right)$$

(7) $$\sigma_{Cu}={\left(\frac{f_{\mathrm{shear}}}{f_{\mathrm{total}}}\right)}^{1/2}\sqrt{\sigma_{\mathrm{coh}}^{2}+\sigma_{\mathrm{mod}}^{2}}+{\left(\frac{f_{\mathrm{loop}}}{f_{\mathrm{total}}}\right)}^{1/2}\sigma_{\mathrm{Orowan}}$$

where *M* and *v* are the Taylor factor and Poisson’s ratio, respectively, *G* is the shear modulus for low-carbon steel, *b* is Burger’s vector taken as 0.2482 nm, 𝑙 is the mean nearest-neighbor particle spacing, $f_{\mathrm{shear}}$ and $f_{\mathrm{loop}}$ are the Cu-rich particle fraction that result from the cutting though and Orowan strengthening mechanisms, respectively, $\sigma_{\mathrm{coh}}$ and $\sigma_{\mathrm{mod}}$ are the coherent misfit strengthening and modulus strengthening effects, respectively. In addition, the precipitation of alloying Fe_3_C was not considered because the mean Fe_3_C size was over 50 nm so the precipitation strengthening was weak.

The tested RA content was about 5.3% in the 0.12C-3.13Mn steel (Figure 6), resulting in the impressive −40 °C impact energy of 179 J after being tempered at 600 °C for 5 h (Figure 4). In our previous study, the small amount of RA could obviously increase the low-temperature toughness by the positive TRIP effect [14,18]. With increasing time of intercritical tempering, the RA content increased but the dislocation density decreased [13]. When the RA content increased, more RA played an important role in improving the low-temperature toughness, resulting in the variation from 133 J to 179 J. However, with further tempering, the increasing RA content and decreasing density of dislocation jointly increased the −40 °C impact energy to 187 J. Different from the variation of the −40 °C impact energy, the strength decreased as the tempering time increased. In addition to RA and dislocation variation, the precipitation coarsening was another reason for the decreased strength (Figure 9a,b). Fortunately, the Orowan coarsening was inhibited by the addition of Mo.

In S4330 steel, there was little RA but plenty of Fe_3_C particles. The large-sized Fe_3_C particles were beneficial to inhibit the dislocation recovery (Figure 9d) and recrystallization (Figure 7c) [39]. It was found that the recrystallization fraction of S4330 steel was limited so that the high-density dislocation was preserved. In addition, the fine grain size and solution C were other reasons for the high strength (Figure 3a). With the tempering time increasing from 3 h to 10 h, the decreased strength (about 70 MPa) was mainly attributed to the decreased dislocation density (Figure 3a). On the contrary, the decreased dislocation density improved the −40 °C impact energy. However, the −40 °C impact energy hardly increased. There were two reasons: on the one hand, the carbides continued to coarsen; on the other hand, the dislocation density decrease was relatively low. Compared to 0.12C-3.13Mn steel, the −40 °C impact energy of S4330 steel was lower than that of 0.12C-3.13Mn steel. However, it should be noted that the effective grain size of S4330 steel was smaller than the latter, and the fraction of high-angle grain boundaries and dislocation density were higher than the latter (Figure 8). The high-angle grain boundary was an effective way to restrain crack propagation by increasing the crack growth path [40]. The result suggested that the role of RA was more important in toughness than ductility. However, it is difficult to estimate the effect of coarse Fe_3_C on toughness.

### 4.3. Fatigue Mechanism Analysis

The fitted FCGR and Δ*K* curves (at the second stage) according to the Paris equation followed:

(8) $${\frac{da}{dN}}_{0.12C-3.13\mathrm{Mn}}=2.218\times 10^{-8}{\left(\Delta K\right)}^{2.550}$$

(9) $${\frac{da}{dN}}_{S4330}=3.833\times 10^{-8}{\left(\Delta K\right)}^{2.392}$$

**Figure 12:**
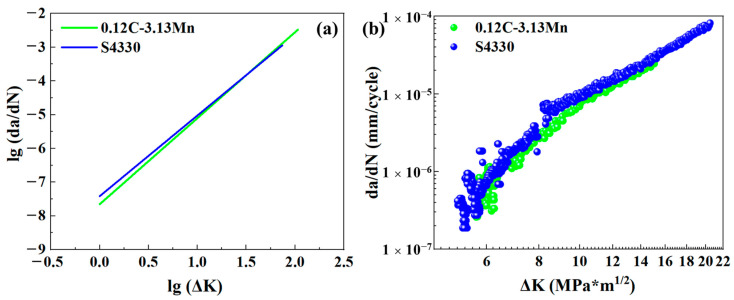
Δ*K_th_* of (**a**) 0.12C-3.13Mn steel and (**b**) S4330 steel.

Based on the fitted result, it was found that 0.12C-3.13Mn steel had a lower C value but a higher m value. From the mathematical point of view, the FCGR was lower for the 0.12C-3.13Mn steel, but the FCGR would increase more rapidly than that of S4330 steel in the second stage of the FCGR curves. Generally, the Δ*K* corresponding to the FCGR (da/dN) of 10^−7^ mm/cycle was regarded as the Δ*K_th_* [41]. Therefore, the estimated Δ*K_th_* of 0.12C-3.13Mn steel and S4330 steel were 1.805 and 1.493 MPa*m^1/2^, respectively (Figure 12a). Evidently, the Δ*K_th_* were underestimated, resulting from the mismatched result by the Paris equation formula fitting. However, this result showed that the Δ*K_th_* of 0.12C-3.13Mn steel was higher than that of S4330 steel, potentially showing a higher capacity to inhibit crack formation [42]. Furthermore, the actual Δ*K_th_* were measured using experiments, as shown in Figure 12b. It was noted that the detected FCGR were all above 10^−7^ mm/cycle. The FCGR curves were fitted using polynomial fit with the order of 2:

(10) $${\frac{da}{dN}}_{0.12C-3.13\mathrm{Mn}}=3.38\times 10^{-6}-1.71\times 10^{-6}\Delta K+2.07*10^{-7}{\left(\Delta K\right)}^{2}$$

(11) $${\frac{da}{dN}}_{0.12C-3.13\mathrm{Mn}}=6.00\times 10^{-6}-2.63\times 10^{-6}\Delta K+2.87*10^{-7}{\left(\Delta K\right)}^{2}$$

The fitting goodness was 0.9954 and 0.9874 for 0.12C-3.13Mn steel and S4330 steel, respectively, showing high fitting accuracy. The calculated Δ*K_th_* for 0.12C-3.13Mn steel and S4330 steel were 5.23 and 4.88 MPa*m^1/2^, respectively. The experimental result showed good agreement with the extension of the Paris equation curve. The higher capacity to inhibit crack formation mainly came from the lower strength (lower dislocation density) and the presence of RA.

**Figure 13:**
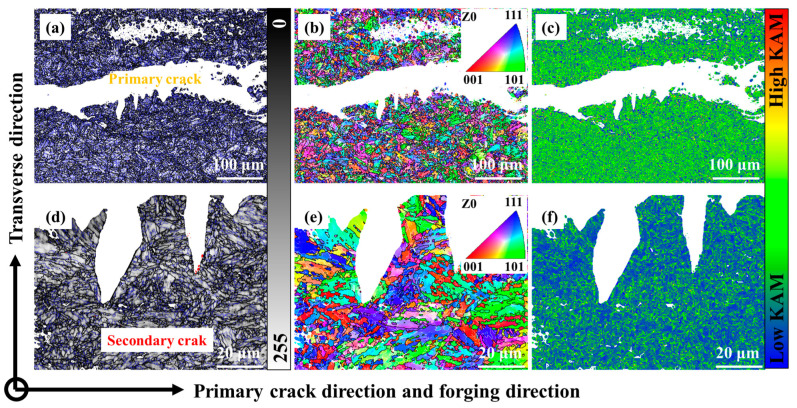
FCG path of 0.12C-3.13Mn steel: (**a**–**c**) Macroscopic morphology; (**d**–**f**) Local magnification region. (**a**,**c**) are the band contrast maps marked by grain boundaries; (**b**,**e**) are the IPF maps; (**c**,**f**) are the KAM maps.

**Figure 14:**
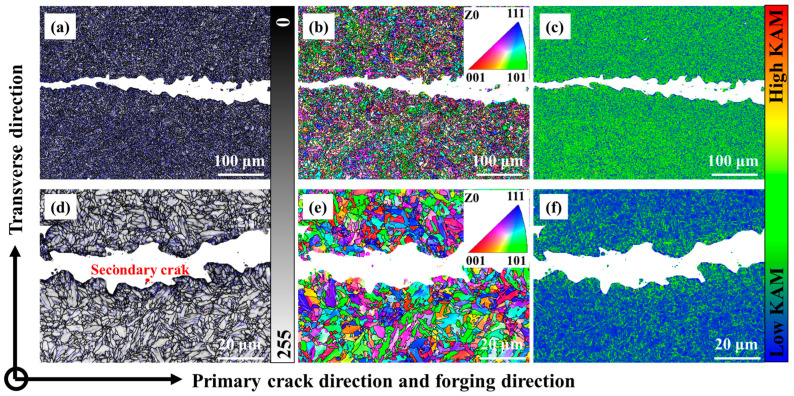
FCG path of S4330 steel: (**a**–**c**) Macroscopic morphology; (**d**–**f**) Local magnification region. (**a**,**c**) are the band contrast maps marked by grain boundaries; (**b**,**e**) are the IPF maps; (**c**,**f**) are the KAM maps.

There were different FCG behaviors of 0.12C-3.13Mn steel and S4330 steel (Figure 10 and Figure 12). The FCG path characterization of these two types of steel are displayed in Figure 13 and Figure 14, respectively. In the 0.12C-3.13Mn steel, the scanning area was close to where the crack started due to the wider primary crack paralleled to the forging direction (Figure 13a,d). However, it was not difficult to find that there were plenty of secondary cracks along the primary crack (Figure 13d). The secondary crack was an intragranular fracture according to the color on the IPF maps (Figure 13b,e). The secondary crack was inhibited when another martensite block was encountered (Figure 13e). The result indicated that the block, surrounded by a high-angle grain boundary, was an effective grain unit to prevent crack propagation [40]. The existence of secondary cracks increased the FCG energy so that the FCG rate was controlled at a low level, showing secondary cracks can inhibit the formation of crack initiation [43]. This explained that the 0.12C-3.13Mn steel had higher threshold value. Furthermore, there was a higher KAM value (local misorientation) near the primary crack, while the KAM value far away from the primary crack was lower (Figure 13c,f), showing a higher stress concentration near the crack. However, it was necessary to recognize that the martensite transformation from RA was dangerous for the crack because the martensite enriched Mn and C had higher hardness as the potential crack source.

Different from that of 0.12C-3.13Mn steel, the scanning area of S4330 steel nearly reached the ending of FCG (Figure 14a,d). Although the counts of secondary cracks were the same as that of 0.12C-3.13Mn steel, the secondary crack length and size was far below that of it (Figure 14), which explained that the FCGR were high as the ΔK increased. Another martensite block could also effectively inhibit the FCG (Figure 14b,e). There was an obvious difference in KAM where the stress concentration range of S4330 steel was lower than that of 0.12C-3.13Mn steel (Figure 14c,f), attributed to the lower stress levels. The finer microstructure of S4330 steel helped to decrease the FCGR, and this phenomenon was more obvious in the late second stage of the FCGR curves. Therefore, it is necessary to refine the microstructure to inhibit the high FCGR in the second and third stages in 0.12C-3.13Mn steel.

## 5. Conclusions

A new-type alloying composition (0.12C-3.13Mn steel) was designed to replace AISI 4330 steel for the more rigorous service environment applied in the oil and gas industry, and the main conclusions are as follows:

(1) The yield strength of 0.12C-3.13Mn steel at 957 MPa was slightly lower than that of S4330 steel at 1060 MPa when subjected to the same tempering process (600 °C for 5 h), but the ductility of the former was superior to that of the latter. The main reason for the lower strength was the low carbon content, dislocation density, and larger effective grain size.
(2) The −40 °C impact energy of 0.12C-3.13Mn steel reached 179 J, which was obviously higher than that of S4330 steel (about 115 J), resulting from the lower dislocation density and the presence of 5.3% RA.
(3) The (V, Mo)C and Cu-rich particles jointly provided a high precipitation strengthening, leading to a higher yield ratio.
(4) The 0.12C-3.13Mn steel showed a higher capacity to inhibit crack initiation than the S4330 steel due to the higher 5.23 MPa*m^1/2^. The FCGR of 0.12C-3.13Mn steel increased more rapidly than S4330 steel, and it was higher than that of S4330 steel at the end of the second stage. However, the FCGR of 0.12C-3.13Mn steel finished at 0.00330 mm/cycle, which was much higher than the latter at 0.00114 mm/cycle. The high tolerance of FCG in 0.12C-3.13Mn steel was due to the presence of RA and secondary cracks.

## Figures and Tables

**Table 1:** Nominal composition of 0.12C-3.13Mn steel and S4330 steel (wt.%).

Samples	C	Si	Mn	Cr	Ni	Mo	V	Cu
0.12C-3.13Mn	0.12	0.20	3.13	1.01	1.90	0.41	0.29	1.0
S4330	0.33	0.25	0.52	1.07	2.95	0.45	0.05	0.03
